# The utility of pelvic lymph node dissection in PSMA-PET negative intermediate and high-risk prostate cancer patients undergoing radical prostatectomy

**DOI:** 10.1007/s00345-026-06330-3

**Published:** 2026-03-11

**Authors:** Giuseppe Reitano, R. Jeffrey Karnes, Giacomo Novara, Daniel S. Roberson, Mohamed E. Ahmed, Filippo Carletti, Umar Ghaffar, Giuseppe Dinoi, Salvatore Carrozza, Maurizio Bentivoglio, Simone Botti, Carlo Prevato, Giovanni Betto, Fabio Zattoni, Fabrizio Dal Moro

**Affiliations:** 1 Department of Surgery, Oncology and Gastroenterology, Università Padova, 35128 Padova, PD Italy; 2 Department of Urology, Mayo Clinic, 55905 Rochester, MN USA; 3 Urology Unit, Azienda Ospedale Università Padova, 35126 Padova, PD Italy

**Keywords:** Prostate cancer, Lymph node dissection, PSMA-PET, Biochemical recurrence

## Abstract

**Purpose:**

To compare perioperative and early oncological outcomes of robotic radical prostatectomy (RP) with and without extended pelvic lymph node dissection (PLND) in a cohort of intermediate (IR)-to-high-risk (HR) prostate cancer (PCa) patients.

**Methods:**

Data was prospectively collected from 88 patients (47 PLND, 41 no-PLND) with unfavorable intermediate- and high-risk miN0M0 PCa, all staged preoperatively with PSMA-PET/CT. Outcomes were assessed using Kaplan-Meier curves, uni-and multivariable Cox regression, for biochemical recurrence-free survival (BCRFS) and biochemical failure-free survival (BCFFS). A propensity score matched analysis with Cox regression was undertaken matching 1:1 for potential confounders (age, iPSA, preoperative ISUP, cT stage at MRI).

**Results:**

Both groups (PLND and no-PLND) had the same proportion of HR patients (*p* = 1.0). The no-PLND group had a shorter median operative time by 50 min (*p* < 0.01). Conversely, the PLND group experienced significantly higher rates of 90-day high-grade complications (*p* = 0.03) and lymphoceles (*p* < 0.01). Over a median 20.5-month follow-up, no significant differences emerged in BCRFS (*p* = 0.59) or BCFFS (*p* = 0.76). Uni- and multivariable analyses adjusted for UCSF CAPRA and CAPRA-S variables, as well as propensity score matching, confirmed PLND was not associated with improved BCFFS. In patients who did recur, the sites of recurrence did not differ between the two groups (*p* = 0.62), with pelvic nodal recurrence being the most common site of recurrence in both groups (6, 50% for PLND and 3, 60% for no-PLND).

**Conclusions:**

In this short-term follow-up, performing PLND increased high-grade postoperative complications without providing a clear early oncological benefit regarding BCRFS, PSA persistence, or recurrence location.

**Supplementary Information:**

The online version contains supplementary material available at 10.1007/s00345-026-06330-3.

## Introduction

Robot-assisted radical prostatectomy (RARP) with pelvic lymph node dissection (PLND) is one of the primary treatment options for patients with intermediate to high-risk (HR) localized prostate cancer (PCa) [1]. Extended PLND (ePLND) provides critical staging information and may be useful to guide postoperative management and risk stratification. However, the therapeutic benefit of PLND is still under debate. Randomized trials have failed till now to demonstrate consistent survival benefit in patients without detectable nodal disease (cN0) [2, 3]. Only one randomized trial showed an improved metastasis-free survival mainly for patients with pathologically positive lymph nodes (pN1) [4]. However, PLND is associated with an increased risk of perioperative complications, including bleeding, lymphocele, thromboembolic complications, and a higher rate of postoperative readmissions, which may impact the overall benefit-risk ratio for patients [5].

Increasing evidence suggests that primary metastatic lymph node sites may lie outside the boundaries of an ePLND, potentially leading to missed nodal metastases both outside and, at times, even within the dissection template [6]. Preoperative nomograms are widely used to evaluate the likelihood of lymph node invasion (LNI) and choose the best candidate for PLND. However, more than half of patients undergoing ePLND based on the 2019 Briganti nomogram result are classified as pN0 at final pathology [7]. Even the new Briganti 2023 nomogram, which has a C-index of 77% for LNI, still exposes many patients with negative pathological nodes to ePLND [8]. As the landscape of PCa staging evolves, novel molecular imaging such as Positron Emission Tomography/Computed Tomography with Prostate-Specific Membrane Antigen (PSMA-PET/CT) have emerged as potential alternatives to conventional imaging (CT scans and bone scans) to improve patient selection before primary treatments. PSMA-PET/CT has demonstrated superior sensitivity and specificity for detecting LNI compared to conventional imaging [9, 10].

Given the lack of confirmed oncologic benefit and the known morbidity, there is growing interest in exploring a PLND-sparing approach. This approach could offer significant benefits in terms of reducing perioperative high-grade complications and hospital stay. To date, no retrospective or prospective studies have evaluated RARP without PLND in HR patients staged with PSMA-PET/CT. To address this gap, our comparative cohort study evaluated early oncological outcomes, perioperative complications, and patterns of recurrence associated with a PLND-sparing approach in IR to HR prostate cancer patients staged with PSMA-PET/CT.

## Materials and methods

### Study population, inclusion and exclusion criteria

Patients with localized HR or unfavorable IR prostate cancer, staged using Ga68-PSMA-PET/CT and treated with primary RARP, with or without ePLND, between July 2022 and July 2024 were identified from two prospectively maintained registries at two academic institutions: Azienda Ospedale-Università Padova (AOUP) and Mayo Clinic. Local IRB authorization for data collection was obtained (Padua IRB number AOP3316, Mayo IRB number 22−010534). This study was performed in accordance with the Declaration of Helsinki.

The decision to perform an ePLND was at the operating surgeon’s discretion. All included patients had an LNI probability exceeding the threshold defined by the Briganti 2019 score [7]. Patients were divided into two cohorts based on whether an ePLND was performed at time of RARP.

Exclusion criteria encompassed patients with clinically positive nodes, suspected distant metastases, locally advanced disease, or those who received neoadjuvant therapies.

### Surgical strategy and ePLND extension

Primary RARP was performed using the Da Vinci X or Xi surgical robot (Intuitive, Sunnyvale, California, USA). An anterior and a Retzius-sparing approach for RARP were used respectively at Mayo Clinic and Padua. Both are high-volume institutions, each performing over 300 RARP annually. When performed, the ePLND included the obturator, internal, and external iliac nodes. Pelvic drainage was not utilized.

### Outcomes

The co-primary outcomes were biochemical failure (BCF)-free survival (defined as the combination of patients with PSA persistence and patients with BCR) and BCR-free survival. BCR was defined as the detection of two consecutive PSA values of 0.2 ng/mL or higher after RARP. BCF was estimated to account also for PSA persistence (considered as early BCR occurring within 2 months after surgery). Secondary endpoints included operative time, estimated blood loss (EBL), 90-day high-grade postoperative complications (≥ 3a), classified according to the Clavien-Dindo system [11], and the incidence of lymphocele within 90 days postoperatively. Patterns of recurrence following RARP, as identified by PSMA-PET imaging, were also analyzed as secondary outcomes.

### Statistical analysis

Continuous variables were summarized using median and interquartile range (IQR), count and percentage described categorical variables. T test and Mann-Whitney U test were used to assess statistical significance among independent groups, Chi-squared test and Fischer exact test were applied when appropriate for categorical variables. Kaplan-Meier curves with Log-rank test were obtained for BCF-free survival and BCR-free survival. Uni- and multivariable Cox regression analysis with hazard ratios (HR) were used to evaluate the role of PLND as a predictor of BCF. Multivariable models were adjusted for predefined clinical and pathological variables based on the UCSF-CAPRA [12, 13] and CAPRA-S scores [14]. The final multivariable model included the primary predictor of interest (PLND) alongside variables that demonstrated statistical significance in the univariable analysis. To confirm the results from the overall cohort, a propensity score matched analysis was undertaken matching 1:1 for potential confounders (age, iPSA, preoperative ISUP, cT stage at MRI). Statistical analyses were performed using R version 4.4.2 (R Project for Statistical Computing; www.r-project.org) and BlueSky version 10.3.4 GA (BlueSky statistics, Chicago, Illinois, USA). Graphical contents were generated using Biorender.com.

## Results

### Baseline and pathological features

A total of 88 patients were included in the study: 41 in the no-PLND group and 47 who underwent ePLND (control group). Baseline characteristics were well balanced and are detailed in the supplementary materials (Supplementary Table S1). HR classification according to EAU criteria was observed in 90.2% of the no-PLND group and 89.4% of the control group (*p* = 1.0). Similarly, baseline Briganti 2019 scores for LNI showed no significant differences between groups (*p* = 0.18).

### Intraoperative endpoints and surgical complications

Operative time was significantly longer in the ePLND group (median 180 min, IQR 155–210) compared to the no-PLND group (median 130 min, IQR 110–160; *p* < 0.01). However, no significant differences were observed in EBL, intraoperative transfusions, or length of hospital stay (Table 1). Pathological stage T3b was more frequently observed in the ePLND group (13 patients, 27.7%) than in the no-PLND group (2 patients, 4.9%; *p* = 0.02). Other pathological characteristics including positive surgical margins, lymphovascular invasion (LVI), and ISUP grade were comparable between the two groups (Table 1). Among patients who underwent ePLND, 10 (21.3%) were classified as pN1. High-grade postoperative complications (Clavien-Dindo ≥ 3a) were rare (6 cases, 6.8%) and occurred exclusively in the ePLND group (*p* = 0.03). Postoperative lymphoceles were also observed only in the control group (9 cases, 10.3%; *p* < 0.01). More details on 0–90 days postoperative complications are available in Supplementary Table S2.

**Table 1:** Operative, Pathological characteristics, and Postoperative complications

	Overall (n=88)	No PLND (n=41)	PLND (n=47)	p-value
*Operative time (min, median, IQR)*	163 (120–180)	130 (110–160)	180 (155–210)	< 0.01*
Blood loss (frequency, %)				
99 ml or less 100–199 ml 200–499 ml 500 ml or above	22 (25.3) 14 (16.1) 35 (40.2) 16 (18.4)	10 (24.4) 7 (17.1) 17 (41.5) 7 (17.1)	12 (26.1) 7 (15.2) 18 (39.1) 9 (19.6)	0.98
Intraoperative transfusions (frequency, %)				
No Yes	84 (96.6) 3 (3.4)	41 (100) 0 (0)	43 (93.5) 3 (6.5)	0.24
Tumor volume (cc, median, IQR)	5 (2.9–7.4.9.4)	5 (3.2–7.7.2.7)	4 (2.4–6.6.4.6)	0.24
Surgical margins (frequency, %)				
Negative Positive	60 (68.2) 28 (31.8)	24 (58.5) 17 (41.5)	36 (76.6) 11 (23.4)	0.07
pT stage (frequency, %)				
T2 T3a T3b	38 (43.2) 35 (39.8) 15 (17)	21 (51.2) 18 (43.9) 2 (4.9)	17 (36.2) 17 (36.2) 13 (27.7)	0.02*
Pathological ISUP (frequency, %)				
1 2 3 4 5	1 (1.1) 8 (9.1) 32 (36.3) 18 (20.5) 29 (33)	0 (0) 4 (9.8) 14 (34.1) 10 (24.4) 13 (31.7)		0.90
Lymphovascular invasion (frequency, %)				
No Yes	67 (76.1) 21 (23.9)	31 (75.6) 10 (24.4)	36 (76.6) 11 (23.4)	0.91
*Length of stay (days, median, IQR)*	1 (1–2)	1 (1–2)	1.5 (1–3)	0.17
High grade postoperative complications 0–90 days (Clavien-Dindo) (frequency, %)				
< 3a ≥ 3a	82 (93.2) 6 (6.8)	41 (100) 0 (0)	41 (87.2) 6 (12.8)	0.03*
Lymphocele (0–90 days) (frequency, %)				
No Yes	78 (89.7) 9 (10.3)	41 (100) 0 (0)	37 (80.4) 9 (19.6)	<0.01*

*IQR* interquartile range, *ISUP* International Society of Urological Pathology

### Follow-up and patterns of recurrence

Median follow-up was 20.5 months (IQR 13–26). During the follow-up period no significant differences were observed between the two groups in terms of recurrence site (Supplementary Table S4, *p* = 0.62), as assessed by molecular imaging.

In the PLND group, among patients with BCF who were re-staged with PSMA-PET, 6 (50%) experienced recurrence in pelvic lymph nodes (external, internal iliac, or obturator; Fig. 2), while 5 (41.7%) had local recurrence. In the no-PLND group, 3 (60%) of patients showed recurrence in pelvic lymph nodes, and 1 (20%) had recurrence in the prostatic bed. Notably, no extra-pelvic (common iliac nodes or above) or distant metastases were identified during follow-up in either group. In the control group, only 4 patients (4.5%) received adjuvant pelvic radiotherapy combined with androgen deprivation therapy (ADT), all of whom had pN1 disease at final pathology. In the no-PLND group, 1 patient (2.4%) underwent adjuvant pelvic radiotherapy with ADT due to multiple risk factors following RARP. Salvage treatments were administered at similar rates in both groups (20% in the no-PLND group [8 patients] vs. 27.9% in the ePLND group [12 patients], *p* = 0.45). Details on the types of salvage treatment are provided in Supplementary Table S5.

### Biochemical recurrence and biochemical failure-free survival

**Fig. 1:**
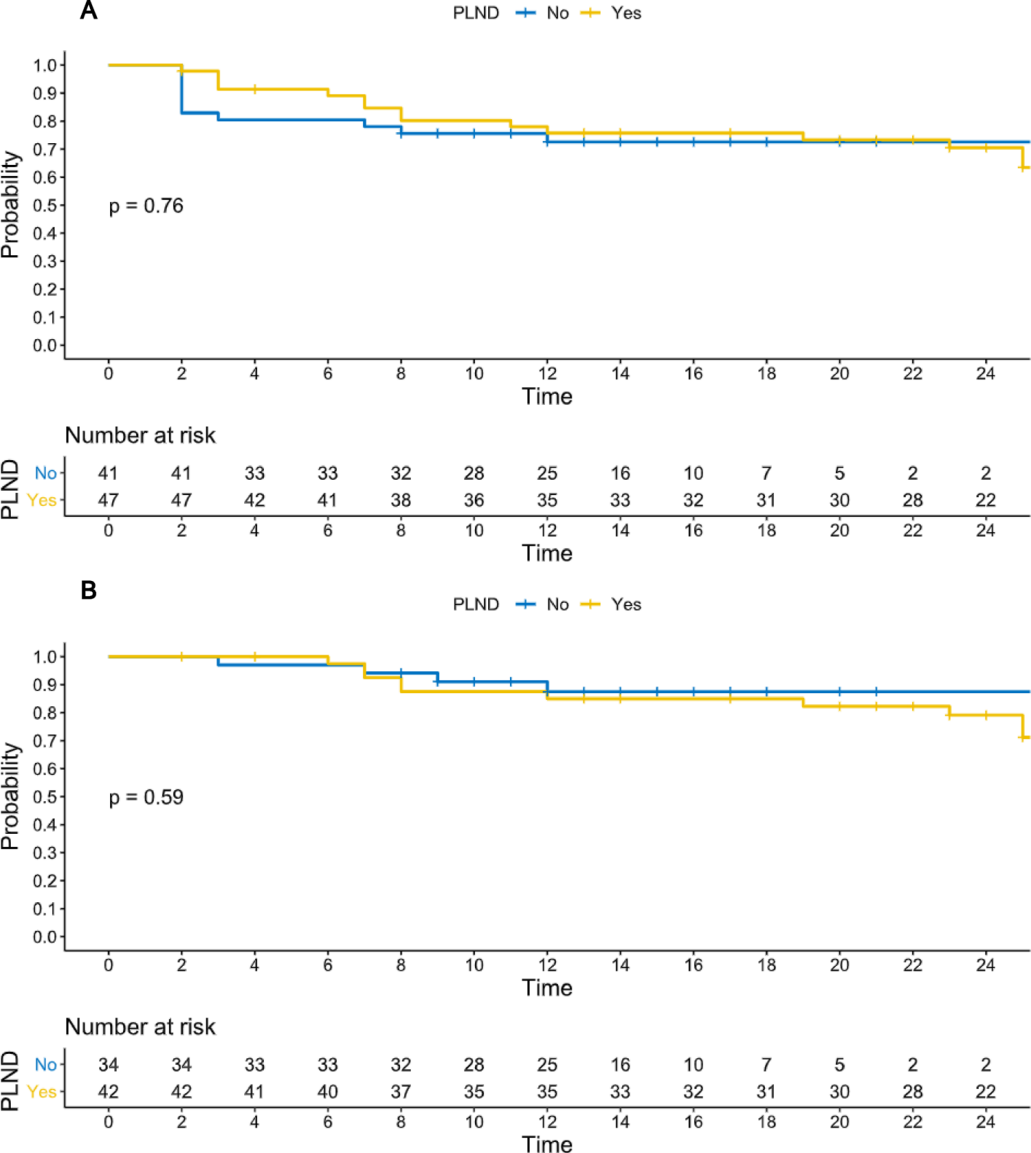
**A** Biochemical failure-free survival; **B** Biochemical recurrence-free survival

No differences in terms of BCF free-survival (*p* = 0.76, Fig. 1A) and BCR-free survival (0.59, Fig. 1B) were seen on Kaplan Meier curves. The univariable Cox regression analysis (Tables 2 and 3) did not show any association between ePLND and BCF (HR 0.86, 95% confidence interval [CI] 0.39–2.0.39.0, *p* = 0.73). Similar results were obtained after adjusting for UCSF-CAPRA variables (HR for PLND 0.83, 95%CI 0.37, 1.9, *p* = 0.61, Table 2) and CAPRA-S variables (HR for PLND 0.7, 95%CI 0.3, 1.8, *p* = 0.44, Table 3). The results from the main analyses were further supported by the propensity score matching analysis (Supplementary Table S6-7).

**Table 2:** Biochemical failure-free survival - Uni and multivariable analysis adjusted for UCSF-CAPRA variables

Parameter	Univariable analysis		Multivariable analysis	
	HR (95% CI)	*p*-value	HR (95% CI)	*p*-value
*PLND*				
No	Ref		Ref	
Yes	0.86 (0.39, 2.0)	0.73	0.83 (0.37, 1.9)	0.66
*Initial PSA*				
0–10 ng/ml	Ref		Ref	
11->20 ng/ml	2.5 (1.1–5.8)	0.03*	2.2 (0.9–5.4)	0.08
*Gleason score pattern 4 or 5 (biopsy)*				
Patterns 3 only or 4/5 in secondary	Ref		-	
Patterns 4/5 in primary	3.5 (0.48–26.48)	0.22	-	
*cT stage*				
T1-T2	Ref		Ref	
T3	3.6 (1.1–12.2)	0.04*	2.6 (0.7–9.2)	0.15
*Percentage of positive cores*				
< 34%	Ref		-	
≥ 34%	2.1 (0.95–4.5)	0.06	-	

**Table 3:** Biochemical failure-free survival - Uni and multivariable analysis adjusted for CAPRA-S variables

Parameter	Univariable analysis		Multivariable analysis	
	HR (95% CI)	*p*-value	HR (95% CI)	*p*-value
*PLND*				
No	Ref		Ref	
Yes	0.86 (0.39, 2.0)	0.73	0.7 (0.3, 1.8)	0.44
*Initial PSA*				
0–10 ng/ml	Ref		Ref	
11->20 ng/ml	2.5 (1.1–5.8)	0.03*	2.6 (1.1, 6.1)	0.03*
*Definitive Gleason score*				
≤ 3	Ref		-	
> 3	1.8 (0.8, 4.1)	0.15	-	
*Surgical margins*				
R0	Ref		Ref	
R1	4.1 (1.9, 9.1)	< 0.001*	3.2 (1.6, 8.2)	0.002*
*ECE*				
No	Ref		-	
Yes	1.7 (0.9–5.0.9.0)	0.09	-	
*SVI*				
No	Ref		Ref	
Yes	2.0 (1.0, 5.4)	0.04*	2.1 (0.8, 5.5)	0.15

## Discussion

PLND still remains the gold standard for nodal staging in patients with PCa undergoing RARP [1]. Several studies have questioned whether the benefits of PLND truly justify its use in all PCa cases at risk for LNI, particularly given the ongoing uncertainty regarding its oncological advantage. An ePLND may be beneficial in extremely selected patients with HR PCa based on the results of the randomized trial by Touijer et al. [4]. However, the trial’s design did not include molecular imaging, which could have allowed better stratification of patients based on clinical nodal involvement, considering that miN1 patients may have a worse prognosis [15].

In 2020, Preisser et al. analyzed data from a multi-institutional database across four European centers, including 520 IR and 187 h patients treated with RP without PLND [16]. In this study, the decision to omit PLND was not preplanned; rather, the decision was based on the individual surgeon’s discretion. No significant differences were observed in 10-year BCR-free survival, metastasis-free survival, or cancer-specific mortality-free survival between patients who underwent PLND and those who did not. With a median follow-up of 33.5 months, this study has some limitations: the Briganti nomogram used did not incorporate MRI data, the proportion of patients who underwent MRI was not reported, and PSMA-PET/CT was not yet utilized at the time of surgery [16]. A recent cohort study conducted in Japan analyzed the 3-year BCR-free survival among patients who underwent PLND versus those who did not. Using a propensity score-matched design, the study found no significant differences in 3-year BCR-free survival between the two groups. This lack of oncological benefit was consistent across both intermediate-risk (IR) and high-risk (HR) patients. Furthermore, a propensity-adjusted multivariate Cox regression analysis did not identify PLND as an independent predictor of oncological outcomes [17]. However, PSMA-PET was not used as a staging tool. Another study conducted at the Martini-Klinik Prostate Cancer Center compared 3-year BCR rates between patients who underwent PLND and those who received RARP alone [18]. All patients had IR PCa and were staged using PSMA-PET. Of the 371 patients included, 333 (90%) underwent PLND, while 38 (10%) did not. Over a median follow-up of 36 months, which represents one of the longest reported to date on this topic, no significant difference in BCR-free survival was observed (78.7% with PLND vs. 76.7% without, *p* = 0.8). The study by Incesu et al. remains the only investigation into a PLND-sparing approach for IR PCa patients in the PSMA-PET era [18]. Our study showed results consistent with those of the three previously mentioned experiences, but in a cohort primarily composed of HR PCa patients (89.8%) staged with PSMA-PET/CT. Notably, PLND did not demonstrate any benefit in terms of PSA persistence or BCR when compared to a PLND-sparing approach. PLND was not a predictor of BCF-free survival in the multivariable Cox regression model and had no impact on BCR-free survival. Furthermore, patterns of short-term recurrence appeared to overlap between the two groups. Kaplan Meier curve for BCR-free survival appeared similar to that shown by the only published study including PSMA-PET [18]. The absence of a detectable difference between the groups at two years, in a sample of mainly HR PCa patients, adds to the existing evidence from previous experiences exploring a PLND-sparing approach in IR and HR populations with longer follow-up [17, 18].

**Fig. 2:**
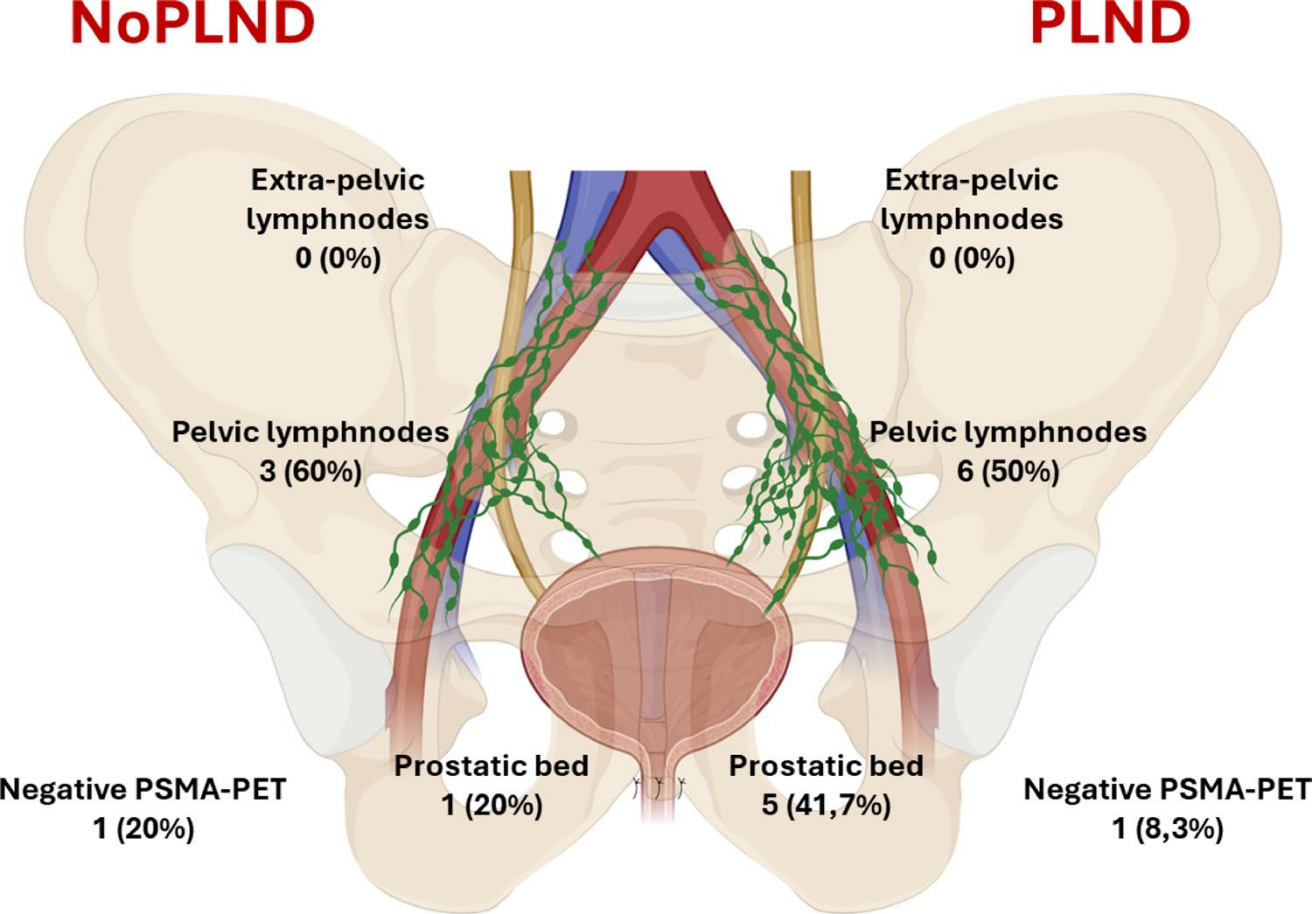
Distribution of recurrences detected with PSMA-PET/CT among patients with biochemical failure treated with pelvic lymph nodes dissection and patients treated with radical prostatectomy alone

In terms of recurrence mapping, pelvic lymph nodes were the most common site of recurrence in our cohort (Fig. 2), raising questions about the actual effectiveness of ePLND. This finding is consistent with the study by Francolini et al., in which pelvic lymph nodes were the most frequent site of recurrence after RP (25%), detected using PSMA-PET/CT. Notably, 80.5% of the patients in the study underwent ePLND [19].

As anticipated, omitting PLND significantly reduced operative time. Moreover, patients in the no-PLND group did not experience any lymphoceles or high-grade complications. Previous studies have also demonstrated reductions in operative time and morbidity associated with a less extensive dissection template [5] with even greater benefits observed when a PLND-sparing approach is adopted [17].

One of the challenges associated with the increasing use of PSMA-PET/CT as a routine staging tool before RARP is the high cost of molecular imaging. However, a recent report found that while PSMA-PET/CT increases costs in both Europe and the USA, the expense of using this advanced imaging technique remains relatively low compared to the potential costs of an inaccurate diagnosis and the unnecessary treatments and complications that may follow [20]. A recent subgroup analysis of a systematic review found that PSMA-PET has a negative predictive value of 81% for LNI and modest sensitivity for patients with HR PCa [10]. Nonetheless, both the available literature and the results from our study suggest that close monitoring, PSMA-PET re-staging, and timely effective salvage treatments may compensate for the lack of initial pathological lymph node staging at the time of RARP.

Our study has some limitations. Firstly, the lack of randomization increases the risk of selection bias. A small sample size affects power and exposes to a risk of not detecting significant differences that may exist in a larger sample. Additionally, RARP was performed by multiple surgeons, with a Retzius-sparing approach used at the Italian center and an anterior approach preferred at the American clinic. The follow-up period was relatively short, though the comparison of PSA persistence rates yielded promising results. Furthermore, the similarity in baseline risk factors (e.g., initial PSA, clinical T stage) and pathological features (particularly ISUP grade, LVI, and positive surgical margin rates), combined with the results of the propensity score matching analysis, may help mitigate potential selection bias. Given the short follow-up, it was not possible to compare the distant metastasis-free survival and the overall survival between the two cohorts. Three ongoing clinical trials are currently investigating PLND-sparing strategies [21–23], two of which incorporate preoperative staging with PSMA-PET [21, 22]. The results of these studies are expected to provide further insights into this topic.

## Conclusions

These findings suggest that PLND is associated with a higher incidence of postoperative complications. Furthermore, a clear benefit in terms of BCR-free or BCF-free survival was not evident when comparing PLND to a PLND-sparing approach in this short follow-up study. Similar recurrence patterns may suggest that, although PLND has long been considered the gold standard for lymph node staging, it may be operator-dependent and not a mandatory part of primary surgical treatment in miN0M0. However, the study’s low power and short follow-up preclude definitive conclusions. Results from ongoing, long-term randomized studies are needed to confirm these findings and assess their implications for clinical practice.

## Supplementary Information

Below is the link to the electronic supplementary material.

Supplementary Material 1

## Data Availability

Data sharing requests should be made to [fabio.zattoni@unipd.it](mailto: fabio.zattoni@unipd.it) for deidentified data. Such requests will be considered by the study team after publication following review and approval of proposals, and with appropriate data-sharing agreements in place.
