# Supplementary material for: The utility of pelvic lymph node dissection in PSMA-PET negative intermediate and high-risk prostate cancer patients undergoing radical prostatectomy
Source: World J Urol. 2026 Mar 11;44(1):231. doi: 10.1007/s00345-026-06330-3 (PMC12979355; doi:10.1007/s00345-026-06330-3)
Supplement: Supplementary file 1 — Supplementary Material 1 [file 345_2026_6330_MOESM1_ESM.docx]

|  | Overall  (n=88) | No PLND  (n=41) | PLND  (n=47) | p-value |
| --- | --- | --- | --- | --- |
| **Age (years, median, IQR)** | 67 (62-71.3) | 68 (63-73) | 66 (61.5-70.5) | 0.23 |
| **BMI (m2/kg median, IQR)** | 27.5 (25.1-29.4) | 27 (24.6-28.4) | 28.3 (25.8-30.4) | 0.02* |
| **ECOG PS**  **(frequency, %)**  0  1  2 | 57 (64.8)  27 (30.7)  4 (4.5) | 23 (56.1)  15 (36.6)  3 (7.3) | 34 (72.3)  12 (25.5)  1 (2.1) | 0.22 |
| **iPSA (mg/dl, median, IQR)** | 8 (5.6-12.2) | 7.4 (5.3-11.3) | 8.4 (5.8-13.9) | 0.31 |
| **cT stage**  **(frequency, %)**  cT1c  cT2  cT3 | 35 (39.8)  49 (56.6)  4 (4.6) | 16 (39)  23 (56.1)  2 (4.9) | 19 (40.4)  26 (55.3)  2 (4.3) | 0.93 |
| **Biopsy ISUP**  **(frequency, %)**  1  2  3  4  5 | 4 (4.6)  5 (5.7)  20 (22.7)  31 (35.2)  28 (31.8) | 1 (2.4)  2 (4.9)  7 (17.1)  18 (43.9)  13 (31.7) | 3 (6.4)  3 (6.4)  13 (27.7)  13 (27.7)  15 (31.9) | 0.49 |
| **Highest PI-RADS score**  **(frequency, %)**  1-2  3  4  5 | 4 (4.8)  2 (2.4)  29 (34)  50 (58.8) | 1 (2.6)  1 (2.6)  16 (41)  21 (53.8) | 3 (6.5)  1 (2.2)  13 (28.3)  29 (63) | 0.75 |
| **PI-RADS maximum diameter (mm, median, IQR)** | 17 (13-21.3) | 15 (12-17) | 19 (14-22.5) | 0.05 |
| **MRI cT stage**  **(frequency, %)**  cT2  cT3a  cT3b | 64 (74.4)  11 (12.8)  11 (12.8) | 35 (87.5)  3 (7.5)  2 (5) | 29 (63)  8 (17.4)  9 (19.6) | 0.03* |
| **EAU Risk Group**  **(frequency, %)**  Intermediate risk  High risk | 9 (10.2)  79 (89.8) | 4 (9.8)  37 (90.2) | 5 (10.6)  42 (89.4) | 1.00 |
| **Briganti 2019** | 16.5 (10.5-38.6) | 13.8 (10-35.9) | 19.3 (10.8-45.2) | 0.18 |

**Supplementary Table S1. baseline features.** IQR = interquartile range; BMI = Body mass index; ECOG PS = Eastern Cooperative Oncology Group Performance Status; PSA = prostate specific antigen; ISUP = International Society of Urological Pathology; PIRADS = Prostate Imaging Reporting And Data System;

|  | Overall  (n=83) | No PLND  (n=40) | PLND  (n=43) |
| --- | --- | --- | --- |
| None  Catheter malfunction  Hematoma  Abdominal pain and small lymphocele (no drainage)  Postoperative pain  Nausea and pain  Paralytic Ileus  UTI and fever  Hematoma and fever  Lymphocele and fever (no drainage)  Percutaneous lymphocele drainage  DVT, PE, ICU admission | 68 (77.5)  2 (2.3)  1 (1.1)  1 (1.1)  1 (1.1)  1 (1.1)  1 (1.1)  4 (4.5)  1 (1.1)  2 (2.3)  5 (5.7)  1 (1.1) | 34 (83)  2 (4.9)  1 (2.4)  0 (0)  0 (0)  1 (2.4)  1 (2.4)  2 (4.9)  0 (0)  0 (0)  0 (0)  0 (0) | 34 (72.4)  0 (0)  0 (0)  1 (2.1)  1 (2.1)  0 (0)  0 (0)  2 (4.3)  1 (2.1)  2 (4.3)  5 (10.6)  1 (2.1) |

**Supplementary Table S2. Type of postoperative complications (0-90days).** DVT = deep vein thrombosis; PE = pulmonary embolism; ICU = intensive care unit

|  | Overall  (n=88) | No PLND  (n=41) | PLND  (n=47) | p-value |
| --- | --- | --- | --- | --- |
| **PSA persistence after surgery**  **(frequency, %)**  No  Yes | 76 (86.4)  12 (13.6) | 34 (82.9)  7 (17.1) | 42 (89.4)  5 (10.6) | 0.38 |
| **Adjuvant pelvic RT + HT**  **(frequency, %)**  No  Yes | 83 (94.3)  5 (5.7) | 40 (97.6)  1 (2.4) | 43 (91.5)  4 (8.5) | 0.37 |
| **BCR (frequency, %)**  No  Yes | 62 (81.6)  14 (18.4) | 30 (88.2)  4 (11.8) | 32 (78.6)  10 (21.4) | 0.24 |
| **PSMA-PET/CT restaging in case of BCR**  **(frequency, %)**  No  Yes | 71 (80.7)  17 (19.3) | 36 (87.8)  5 (12.2) | 35 (74.5) 12 (25.5) | 0.175 |
| **Site of positive PSMA**  **(frequency, %)**  Negative  Prostate bed  Pelvic lymph nodes | 2 (11.8)  6 (35.3)  9 (52.9) | 1 (20)  1 (20)  3 (60) | 1 (8.3)  5 (41.7)  6 (50) | 0.62 |
| **Salvage treatment**  **(frequency, %)**  No  Yes | 63 (75.9)  20 (24.1) | 32 (80)  8 (20) | 31 (72.1)  12 (27.9) | 0.45 |
| **PSA at last follow-up**  **(ng/ml, median, IQR)** | 0.02 (0.009-0.09) | 0.01 (0.008-0.08) | 0.09 (0.01-0.09) | 0.04* |

**Supplementary Table S3. Oncological follow-up.** IQR = interquartile range; PSA = prostate specific antigen; RT = Radiotherapy; HT = Hormone therapy; BCR = Biochemical recurrence; PSMA-PET/CT = Prostate Specific Membrane Antigen - Positron Emission Tomography/Computed Tomography

| **PSMA/PET uptake** | **No PLND** | **PLND** | **p- value** |
| --- | --- | --- | --- |
| Negative | 1 (20) | 1 (8.3) | 0.62 |
| Prostate bed | 1 (20) | 5 (41.7) |  |
| Pelvic lymph nodes | 3 (60) | 6 (50) |  |

**Supplementary Table S4. Fischer exact test contingency table for PSMA uptake after recurrence.**

|  | Overall  (n=83) | No PLND  (n=40) | PLND  (n=43) |
| --- | --- | --- | --- |
| None  Pelvic RT  Pelvic RT + LHRHa  Pelvic RT + Abiraterone + LHRHa  LHRHa  LHRHa + Local cryoablation | 63 (75.9)  10 (12)  5 (6)  2 (2.5)  2 (2.5)  1 (1.1) | 32 (80)  7 (17.5)  1 (2.5)  0 (0)  0 (0)  0 (0) | 31 (72.1)  3 (7)  4 (9.3)  2 (4.7)  2 (4.7)  1 (2.3) |

**Supplementary Table S5. Details on salvage treatments used (frequency, %).** RT = radiotherapy; HT = hormone therapy; LHRHa = luteinizing hormone-releasing hormone agonists

| **Variabile** | **No PLND (n=38)** | **PLND (n=38)** | **p-value** |
| --- | --- | --- | --- |
| **Operative time (median, min, IQR)** | 135 (116.3-163.8) | 180 (150.0,210) | **<0.001*** |
| **Intraoperative transfusions (frequency,%)** | 0 (0) | 3 (8.1) | 0.229 |
| **Tumor volume (cc, median, IQR)** | 4.9 (3.3, 7.6) | 4.0 (2.4, 6.0) | 0.202 |
| **Positive surgical margins** | 15 (39.5) | 9 (23.7) | 0.217 |
| **pT stage (frequency, %)** |  |  | **0.015*** |
| **pT2** | 19 (50.0) | 11 (28.9) |  |
| **pT3a** | 17 (44.7) | 16 (42.1) |  |
| **pT3b** | 2 (5.3) | 11 (28.9) |  |
| **Pathological ISUP (frequency, %)** |  |  | 0.751 |
| **1** | 0 (0.0) | 1 (2.6) |  |
| **2** | 3 (7.9) | 4 (10.5) |  |
| **3** | 14 (36.8) | 16 (42.1) |  |
| **4** | 9 (23.7) | 6 (15.8) |  |
| **5** | 12 (31.6) | 11 (28.9) |  |
| **Lymphovascular invasion (frequency, %)** | 8 (21.1) | 9 (23.7) | 1.000 |
| **Length of stay (days, median, IQR)** | 1 (1, 2) | 1 (1, 3) | 0.445 |
| **Lymphocele (0-90 days)** | 0 (0) | 5 (13.5) | 0.060 |

**Supplementary Table S6. Propensity score matched analysis**

| **Variable** | **HR (95%CI)** | **p-value** |
| --- | --- | --- |
| PLND | 0.95 (0.37, 2.44) | 0.92 |

**Supplementary table S7. Propensity score adjusted univariable cox regression for BCF**
